# In Vitro Fracture Strength of Teeth Restored with Lithium Disilicate Onlays with and without Fiber Post Build-Up

**DOI:** 10.3390/dj6030035

**Published:** 2018-07-23

**Authors:** Nicola Mobilio, Alberto Fasiol, Francesco Mollica, Santo Catapano

**Affiliations:** 1 Department of Prosthodontics, Dental School, Dental Clinic, University of Ferrara, 44121 Ferrara, Italy; alberto.fasiol@unife.it (A.F.); cts@unife.it (S.C.); 2 Department of Medical Biotechnologies, University of Siena, 53100 Siena, Italy; 3 Department of Engineering, University of Ferrara, 44124 Ferrara, Italy; francesco.mollica@unife.it

**Keywords:** all-ceramic, ferrule effect, fracture test, lithium disilicate, onlays, build-up, post-and-core build-up

## Abstract

To our knowledge there is no data about the mechanical performance of indirect restoration adhesively cemented on teeth without an adequate build-up to provide the correct geometrical configuration. The aim of this study was to compare the fracture strength of human teeth restored with lithium disilicate onlays, with and without fiber post build-up. Methods: Twenty human mandibular molars were horizontally sectioned and divided into two groups (*n* = 10). No treatment was applied in group A. Teeth in group B were endodontically treated, built-up using fiber post and composite core and prepared with a circumferential chamfer providing a 1 mm circumferential ferrule. Lithium disilicate onlays were pressed and luted on teeth using dual-curing luting composite. Teeth were tested under static load. Failures were classified as restorable or not restorable. Failure loads were analyzed with one-way analysis of variance. Failure modes were compared using Pearson’s Chi-square tests. Results: The mean fracture loads were 1383.5 N for group A and 1286.3 N for group B. No difference was found (*p* = 0.6). Ninety per cent of fractures were classified as not restorable in both groups, with no difference (*p* = 0.8). Conclusions: For teeth restored with adhesive procedures and lithium disilicate onlays, the presence of build-up with fiber post to provide retention and resistance form does not influence the fracture strength.

## 1. Introduction

**Figure 1:**
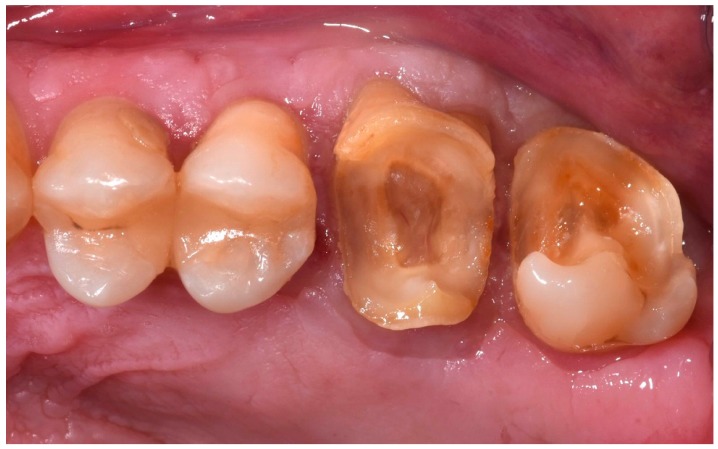
Upper molars with great loss of the coronal structure but yet, vital.

The presence of extensive carious lesions, unsatisfactory restorations, and tooth fractures has resulted in controversy regarding the optimal restorative procedure [1]. When the entire anatomical crown of the tooth is compromised and needs to be covered (Figure 1), the traditional protocol builds up the crown using a root post and then covers it with an indirect restoration (prosthetic crown or onlay). Such a treatment has been considered mandatory to provide an adequate geometrical configuration to allow the tooth to be restored—specifically, the retention and resistance form and ferrule effect, a requirement for the biomechanical success of the restored tooth [2,3,4,5]. This procedure calls for endodontic treatment of teeth that otherwise could be kept vital. An alternative approach may be the cementation of the indirect coverage on the remaining tooth in a “flat-to-flat” mode [6]. In previous years, adhesively luted indirect restorations have been increasingly proposed for prosthetic treatment. At the same time, minimally invasive approaches have become popular, leading to tooth preparations that do not have traditional geometrical characteristics. Studies are needed that investigate the adhesive interface and the new “system” tooth adhesive restoration and to compare them with the traditional prosthetic concept. Under this view, all-ceramic materials represent the ideal choice; they may be adhesively luted on the tooth, allowing perfect integration with dental tissues [7]. No data are available in the literature regarding the mechanical performance of indirect adhesive restoration cemented on teeth without an adequate build-up to provide the correct geometrical configuration.

The objective of the present study was to compare the fracture resistance and failure mode of human teeth restored with lithium disilicate onlay restorations, with and without fiber post build-up. The tested null hypothesis was that teeth restored with and without fiber post build-up would show a similar fracture strength. 

## 2. Results

The means and standard deviations of failure loads are shown in Table 1. 

**Figure 2:**
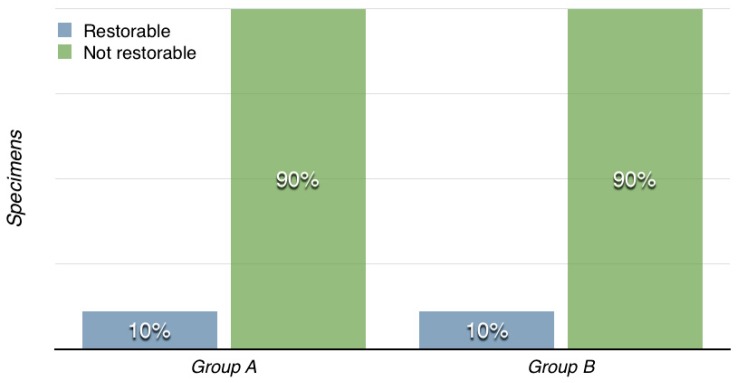
Failure mode distribution.

No significance differences were found between the groups (*p* = 0.6). The failure mode distribution is shown in Figure 2.

**Figure 3:**
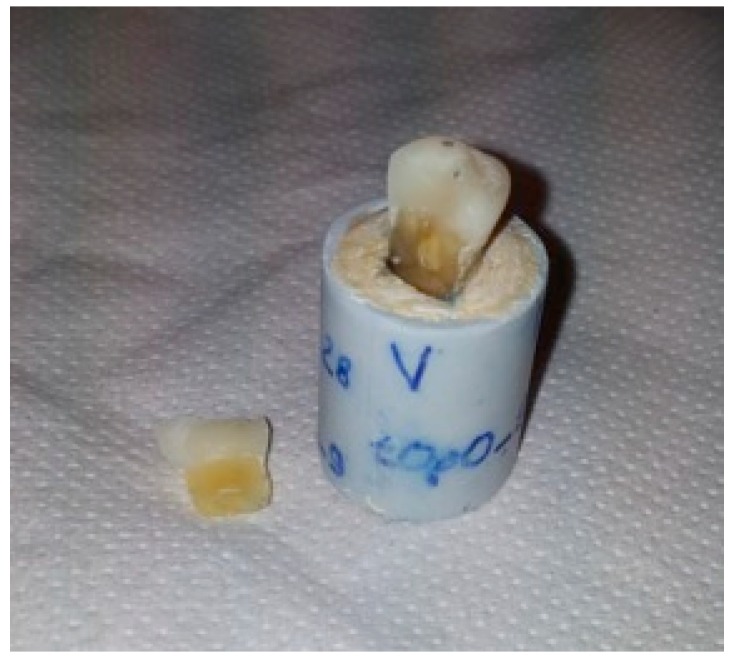
Typical fracture of a specimen from group A after the test.

**Figure 4:**
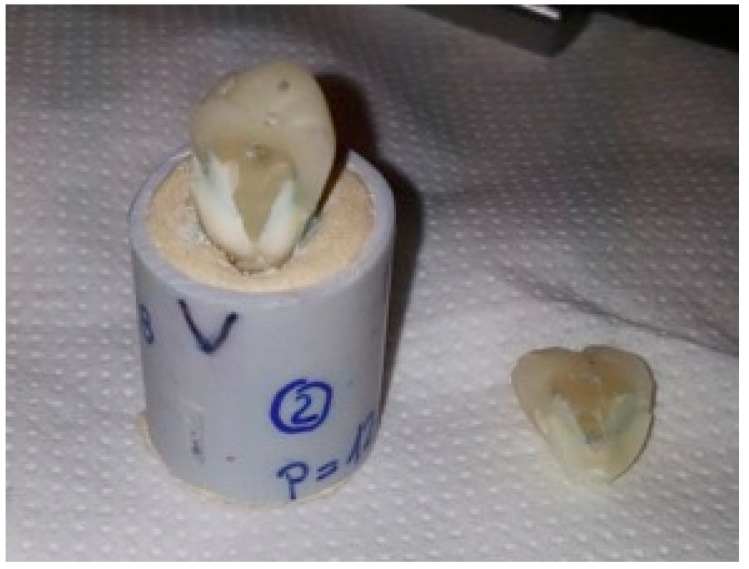
Typical fracture of a specimen from group B after the test.

**Figure 5:**
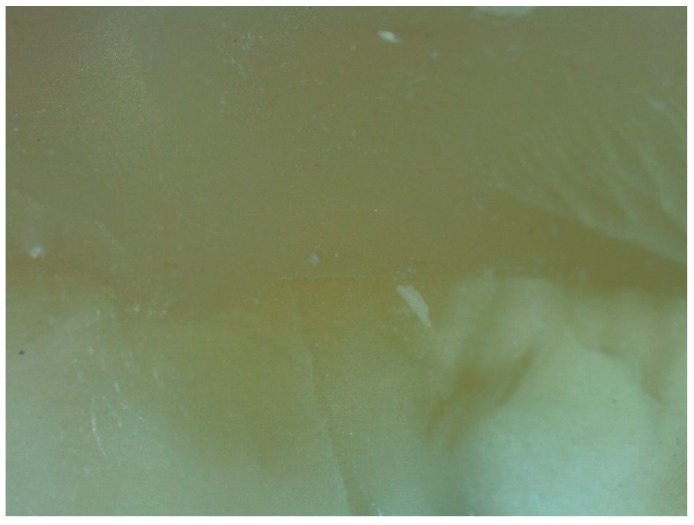
Optical microscope analysis of a specimen from group A after the test.

**Figure 6:**
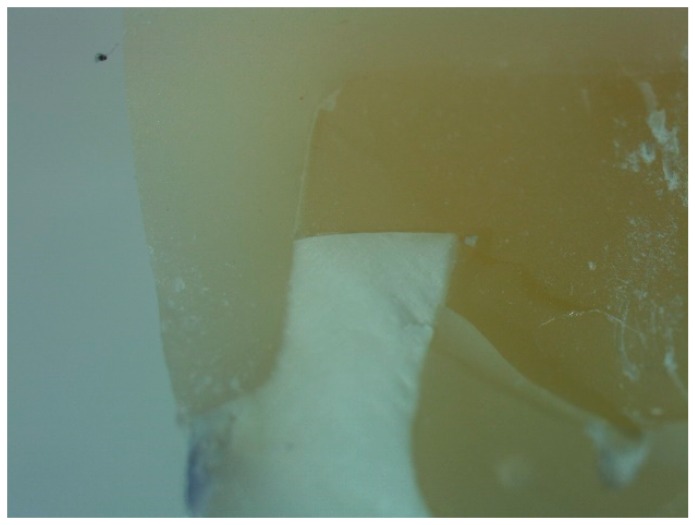
Optical microscope analysis of a specimen from group B after the test.

No significance differences were found (*p* = 0.8). The null hypothesis was accepted. Figure 3 and Figure 4 show typical fractures of specimens in group A and group B, respectively. Figure 5 and Figure 6 show magnifications of the same fractures.

## 3. Discussion

The ferrule effect is traditionally recognized as being mandatory to provide the biomechanical resistance of restored teeth [2,3,5,8]. If ferrule cannot be achieved, the tooth is often defined as not restorable [4]. Such an assumption derives from the classical principles of retention and resistance form that a tooth needs for the success of restoration. Numerous in vitro studies have repeatedly shown the protective role of ferrule in restored teeth [9,10,11,12,13,14,15,16,17]. A recent meta-analysis found that the absence of coronal structure and ferrule might increase the risk of failure of post and core restorations [18]. Another systematic review reached the same conclusion [19]. However, even if these principles have always been valid for restorations delivered with traditional, non-adhesive cements, are they still needed when adhesive luting procedures are applied? Growing evidence suggests that adhesively luting ceramics can restore teeth not only anatomically but also structurally [7,20]. Of course, a real adhesive cementation is possible only with an etchable ceramic, like lithium disilicate. This is the reason why such a material was chosen for the production of indirect restorations. Lithium disilicate is an all-ceramic material with good mechanical aesthetic properties. Compared to other glass ceramics, it shows very good results. Indications for this ceramic are single crowns, veneers and onlays [21,22,23]. Despite its good mechanical performance, lithium disilicate remains a glass ceramic in nature, so it may be etched by using hydrofluoridric acid. Etching the intaglio surface dramatically enhances the adhesion to dental tissues by using resin cement. Strictly following the luting procedures is crucial for the production of the adhesive interface [7,24,25] and to improve the survival of the restorations [26]. In the present study, an oblique, static load was applied on restored teeth until fracture. Oblique loads are the worst for teeth to sustain, and it was chosen to stress the system [27]. The results showed that there was no difference in fracture resistance between the “traditional” group (with a build-up with a post and ferrule effect) and the “new” group with no post and no ferrule. In the latter group, the adhesive interface absorbed the entire load, and it appeared to face it like the group with geometrical preparation. These results confirmed those of previous studies: the ceramic restoration, etched and cemented by luting composite cement, becomes part of the tooth, showing the same biomechanical behavior of intact teeth [7,20]. Furthermore, the mean values of failure loads that the specimens sustained greatly exceeded the bite force registered in experimental studies [28,29]. A recent review found that the presence of a ferrule leads to more favourable fracture patterns [5]. Such a finding was not confirmed by the present results; the distribution of failure mode showed that the restorability does not depend on the preparation of the tooth. The majority of the failures were not restorable, probably because of the high load.

**Figure 7:**
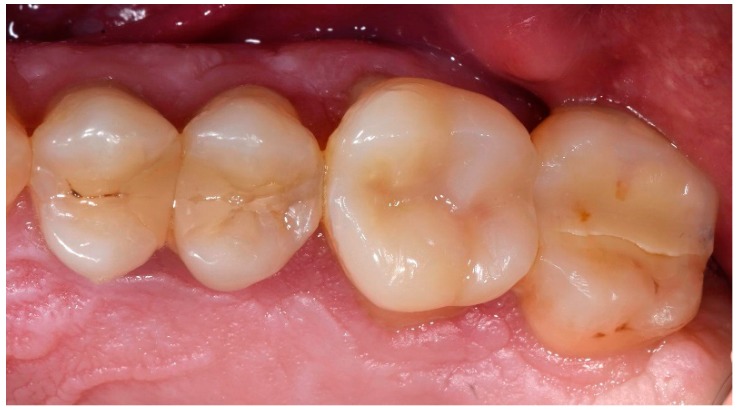
Final restoration of teeth using adhesively luted disilicate onlays without build-up.

Within the limits of the study, the present results are potentially ground-breaking; they demonstrate that, if adequate adhesive procedures are applied (using etching ceramics like lithium disilicate), traditional concepts of tooth preparation (retention form, resistance form, ferrule effect) may be no longer required for the mechanical resistance of restored teeth. This has fundamental clinical consequences; in case of severely compromised but yet vital teeth, endodontic treatment and build-up could be no longer required. So, these teeth could be kept vital, with a fundamental biological advantage and an improved prognosis (Figure 7) [30]. Nevertheless, the biological and economical costs of the rehabilitation may be reduced. 

Some limits of the present study need to be discussed. First of all, there was a limited number of specimens. It is possible that by increasing the number of specimens, different results would be found. Furthermore, the specimens were not submitted to thermocycling before failure loading; thus, different results could be expected due to the potential degradation of the cement interface. Thus, further studies are needed to investigate these possibilities and confirm (or reject) the presented results. 

## 4. Materials and Methods

**Figure 8:**
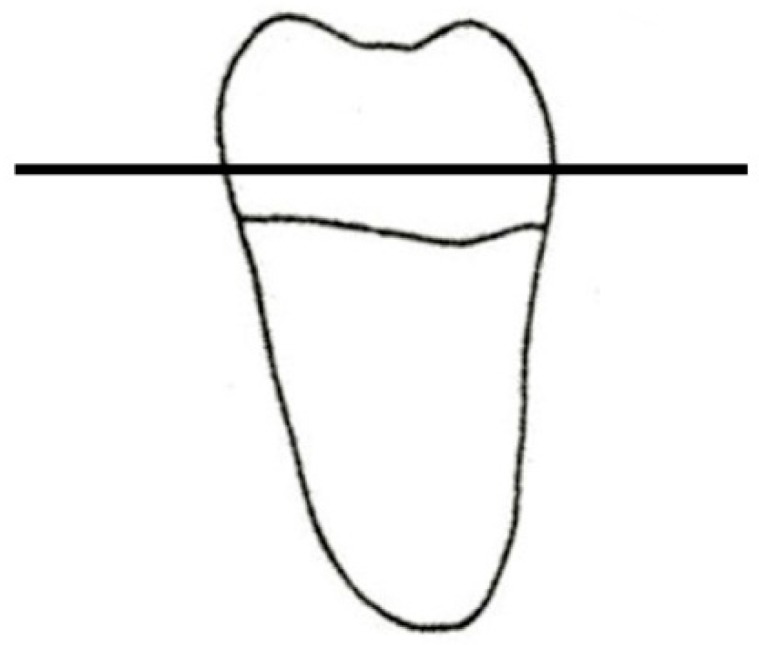
Schematic illustration of sectioning of teeth.

Twenty mandibular molars, extracted for periodontal reasons, were collected after excluding teeth with caries and/or previous restorations. Only teeth with an average buccal–lingual dimension of 10 ± 1 mm and a mesial–distal dimension of 11 ± 1 mm were chosen. After removing dental plaque, calculus, and periodontal tissues with ultrasonic instruments and curettes, the teeth were stored in physiological solution until further use. Teeth were horizontally sectioned 2 mm above the cement–enamel junction (CEJ) using a diamond disk, as illustrated in Figure 8. Teeth were then randomly divided into two groups (*n* = 10): one group (group A) was not built-up and one group was built-up (group B). No treatment was applied on teeth in group A. 

**Figure 9:**
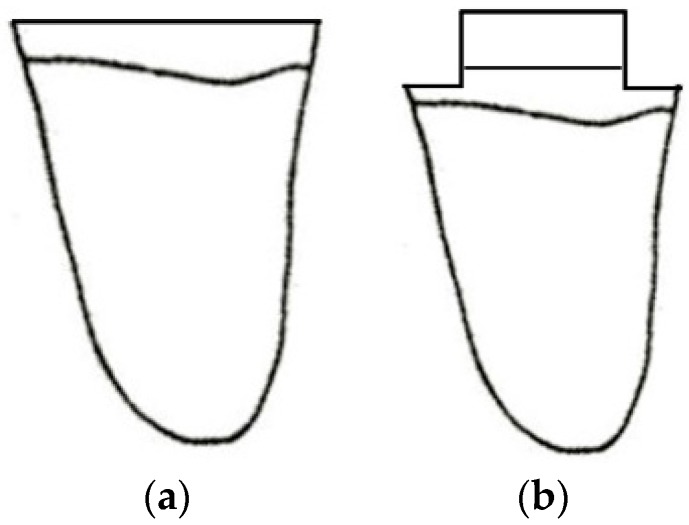
Schematic illustration (distal view) of teeth of both groups before onlay cementation. (**a**): group A; (**b**): group B.

Teeth in group B were endodontically treated. The roof of the pulp chamber was removed, and the canal length was established by placing a #15 K-file (Dentsply Maillefer, Ballaigues, Switzerland) in the root canal until its tip was visible from the apical foramen. The working length was established to be 1 mm shorter. All root canals were prepared to size 30 using manual K-files (Dentsply Maillefer, Ballaigues, Switzerland) and rotary Ni-Ti instruments (ProTaper Universal, Dentsply Maillefer, Ballaigues, Switzerland). Instrumentation was used according to the manufacturers’ instructions. During instrumentation, root canals were irrigated with 5.25% sodium hypochlorite at 37 °C and 10% ethylenediamine tetraacetic acid solution. After preparation, canals were obturated using the vertical condensation technique with warm gutta-percha and a root canal sealer (AH Plus, Dentsply). Then gutta-percha was progressively removed using a handpiece and drill No. 2 (3M ESPE AG, Seefeld, Germany) until 5 mm to the apical foramen. A translucent glass fiber post (GFP; Size #2 RelyX Fiber Post, 3M ESPE AG, Germany) was inserted into the canal of the distal root and cut to the adequate length with a diamond bur to cover it occlusally with 1 mm of resin composite. Posts were carefully cleaned with ethanol and dried with air free of water and oil. A dual-curing luting composite (Multilink Automix, Ivoclar Vivadent AG, Schaan, Liechtenstein) was applied into the root canal in an apical–coronal direction using a tip. Posts were seated into the root canals, and excess cement was removed. Resin cement was light-cured through the coronal portion of the post for 40 s using a halogen curing light (Optilux 501, SDS/Kerr, Danbury, CT, USA). Excess cement was removed using an Arkansas bur mounted on a handpiece. After etching enamel for 30 s and dentin for 10 s with a 37% orthophosphoric acid, a primer (Optibond FL, Kerr, USA) was applied with a clean microbrush and gently dried. Then, a bonding resin (Optibond FL, Kerr, USA) was applied and light-cured for 20 s. The core was built-up using a microhybrid resin composite (Enamel Plus HFO, Micerium). The core was 2 mm high and occupied the central part of the tooth. Thereafter, each tooth was prepared with a 1 mm circumferential chamfer, 1 mm below the core build-up and 1 mm above the CEJ. In this way, a 1 mm circumferential ferrule was provided (Figure 9).

Each tooth from both groups was embedded in a self-curing acrylic resin block (ProBase, Ivoclar Vivadent AG, Schaan, Liechtenstein) in a stainless steel cylinder, up to 2 mm below the CEJ and with its long axis perpendicular to the base of the block.

An onlay was waxed on each tooth and then hot pressed using lithium disilicate ceramic (IPS e.max PRESS, Ivoclar Vivadent AG, Schaan, Liechtenstein). The onlay was 3 mm high and had the same occlusal anatomy for all the teeth. The spacer was applied according to the manufacturer’s instructions. For cementation, a dual-curing luting composite (Multilink Automix, Ivoclar Vivadent AG, Schaan, Liechtenstein) was used. The cementation procedures followed the manufactur’s instructions. The intaglio surface of the ceramic onlays were etched with 5% hydrofluoridric acid (IPS Ceramic gel, Ivoclar Vivadent AG, Schaan, Liechtenstein) for 20 seconds and then rinsed and cleaned for ten minutes in pure alcohol in an ultrasonic bath. Thus, the surface was treated with a universal primer (Monobond Plus, Ivoclar Vivadent AG, Schaan, Liechtenstein) for 60 s and then dried with hot air for another 60 s. The teeth were cleaned and dried, and adhesive (Multilink Primer, Ivoclar Vivadent AG, Schaan, Liechtenstein) was brushed on them for 30 s and dried for 10 s. The cement was applied onto the intaglio surface of the onlays, and then they were seated onto the prepared teeth until the end of polymerization. Teeth were stored for 7 days in physiologic solution at 37 °C.

**Figure 10:**
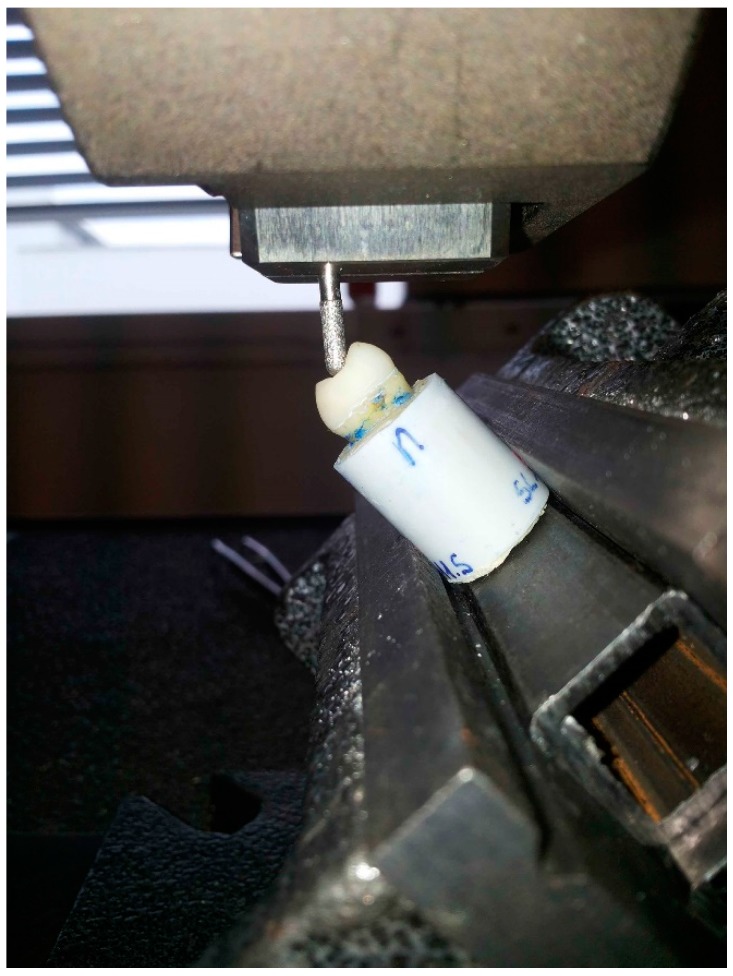
A specimen during the static oblique load in the universal testing machine.

Both groups were tested under compressive load until fracture on a universal testing machine at 45° on the static cusp, with a crosshead speed of 1 mm/min (Figure 10). The value in Newtons (N) at failure was recorded and assumed to be the fracture load. The failures were classified as restorable or not restorable, according to whether it was confined to the restoration or extended to the dental tissues, respectively.

Failure loads in Newtons (N) and failure modes were statistically analyzed (SPSS software v.22 for Mac OSX, IBM, Armonk, NY, USA). The Kolmogorov–Smirnov test was used to confirm the normal distribution of data. Levene’s test was used to confirm the homogeneity of variances. Failure load data were analyzed with one-way analysis of variance (ANOVA). Failure modes were compared using Pearson’s Chi-square tests. The level of significance was set at 0.05.

## 5. Conclusions

Within the limits of this in vitro study, for teeth restored with adhesive procedures and lithium disilicate onlays, the presence of build-up with a root post to provide retention and resistance form did not influence the failure load and mode under a fracture test.

## Figures and Tables

**Table 1:** Means and standard deviations of failure loads (Newtons).

Groups	Mean (Standard Deviation)
Group A	1383.5 (359.4)
Group B	1286.3 (524.8)
